# Modulation of Serotonin Transporter Function during Fetal Development Causes Dilated Heart Cardiomyopathy and Lifelong Behavioral Abnormalities

**DOI:** 10.1371/journal.pone.0002782

**Published:** 2008-07-23

**Authors:** Cornelle W. Noorlander, Frederique F. T. Ververs, Peter G. J. Nikkels, Cees J. A. van Echteld, Gerard H. A. Visser, Marten P. Smidt

**Affiliations:** 1 Rudolf Magnus Institute of Neuroscience, Department of Neuroscience and Pharmacology, University Medical Center Utrecht, Utrecht, The Netherlands; 2 Department of Pharmacy, University Medical Center Utrecht, Utrecht, The Netherlands; 3 Department of Pathology, University Medical Center Utrecht, Utrecht, The Netherlands; 4 Department of Cardiology, University Medical Center Utrecht, Utrecht, The Netherlands; 5 Department of Obstetrics, Neonatology and Gynaecology, University Medical Center Utrecht, Utrecht, The Netherlands; Minnesota State University Mankato, United States of America

## Abstract

**Background:**

Women are at great risk for mood and anxiety disorders during their childbearing years and may become pregnant while taking antidepressant drugs. In the treatment of depression and anxiety disorders, selective serotonin reuptake inhibitors (SSRIs) are the most frequently prescribed drugs, while it is largely unknown whether this medication affects the development of the central nervous system of the fetus. The possible effects are the product of placental transfer efficiency, time of administration and dose of the respective SSRI.

**Methodology/Principal Findings:**

In order to attain this information we have setup a study in which these parameters were measured and the consequences in terms of physiology and behavior are mapped. The placental transfer of fluoxetine and fluvoxamine, two commonly used SSRIs, was similar between mouse and human, indicating that the fetal exposure of these SSRIs in mice is comparable with the human situation. Fluvoxamine displayed a relatively low placental transfer, while fluoxetine showed a relatively high placental transfer. Using clinical doses of fluoxetine the mortality of the offspring increased dramatically, whereas the mortality was unaffected after fluvoxamine exposure. The majority of the fluoxetine-exposed offspring died postnatally of severe heart failure caused by dilated cardiomyopathy. Molecular analysis of fluoxetine-exposed offspring showed long-term alterations in serotonin transporter levels in the raphe nucleus. Furthermore, prenatal fluoxetine exposure resulted in depressive- and anxiety-related behavior in adult mice. In contrast, fluvoxamine-exposed mice did not show alterations in behavior and serotonin transporter levels. Decreasing the dose of fluoxetine resulted in higher survival rates and less dramatic effects on the long-term behavior in the offspring.

**Conclusions:**

These results indicate that prenatal fluoxetine exposure affects fetal development, resulting in cardiomyopathy and a higher vulnerability to affective disorders in a dose-dependent manner.

## Introduction

Mood and anxiety disorders such as depression, panic disorder and obsessive-compulsive disorder are common in women during their childbearing years [1], [2]. The prevalence of depression has been reported to be between 10% and 16% during pregnancy and is becoming a major health issue [3], [4]. In the treatment of depression and anxiety disorders during pregnancy, selective serotonin reuptake inhibitors (SSRIs) are the most frequently prescribed drugs nowadays. SSRIs, like fluoxetine and fluvoxamine, inhibit the reuptake of serotonin (5-hydroxytryptamine or 5-HT) into the presynaptic neuron by binding to the serotonin transporter (5-HTT), which results in an increase of synaptic serotonin levels. SSRIs have no effects on other monoamine transporters, which differentiates them from the previously prescribed tricyclic antidepressants. SSRIs are considered much safer than tricyclic antidepressants, since the toxic dose threshold is much higher and they are believed to have fewer and weaker side effects. Nevertheless, there is still uncertainty concerning the safety of the offspring after antidepressant exposure during pregnancy. Although several studies have reported no associations between congenital malformations and prenatal SSRI exposure, it has been recently shown that fetal exposure to SSRIs results in an increased risk of adverse neonatal effects, including neurological abnormalities, cardiac malformations and persistent pulmonary hypertension [5]–[11]. Furthermore, lower birth weight and an increased risk of preterm birth have been observed after prenatal SSRI treatment [12], [13]. However, it is unknown whether this medication affects the development of the central nervous system of the fetus. Therefore, we have setup a study design to evaluate prenatal SSRI exposure on fetal development and the long-term consequences in terms of behavioral pathology. In the mouse model in which clinical doses were applied to the mothers during pregnancy, we found that the offspring suffered in a dose-dependent manner from a severe form of dilated cardiomyopathy and that surviving mice had severe behavioral abnormalities which may relate to anxiety disorders.

## Materials and Methods

### Animals

Pregnant C57Bl/6-JIco mice (Charles River Laboratory, France) were housed individually on day 6 of pregnancy. Pregnancy was determined by observation of a vaginal plug. The plug date is considered to be day 0 of gestation (embryonic day 0 (E0)). From day 8 until day 18 of pregnancy, mice were injected intraperitonally with either fluoxetine (0.3, 0.6, 0.8 mg/kg/day), fluvoxamine (4.2 mg/kg/day) or with equal volumes of sterile saline. Mice were allowed ad libitum access to food and water. Light/dark cycle (dark phase 19:00–07:00 h), temperature (21°C) and humidity (60%) were kept constant. In cross fostering experiments, pups were placed with another mother a few hours after birth. Offspring was studied at E18, postnatal day 3 (P3), P20 and adult stage (P90). For the adult stage, pups were weaned at P25 and remained group-housed (2–4 animals per cage) with same-sex littermates until experimentation at adulthood. All experiments were approved by the Animal Ethics Committee of the University Medical Center Utrecht and were conducted in agreement with Dutch laws (Wet op de Dierproeven, 1996) and European regulations (Guideline 86/609/EEC).

### High Performance Liquid Chromatography Analysis

For determining placental transfer of SSRIs, pregnant mice were sacrificed at E16 by decapitation five hours after drug administration. Maternal blood was collected and embryos were quickly removed. Embryonic tissue was homogenized with 1 ml of 0.9% NaCl. Maternal plasma and embryonic tissue was stored at −20°C until analysis. For determining placental transfer of SSRIs in humans, maternal blood was obtained during delivery at the end stage of labour of woman who used antidepressants throughout pregnancy. Venous cord blood serum was obtained within the first hour after delivery. Samples were stored at −20°C till analysis.

Fluoxetine and fluvoxamine concentrations were determined by a high-performance liquid chromatography (HPLC) method with UV/Fluorescence detection. Desmethylmaprotyline was used as internal standard. The analytes were extracted from plasma and embryonic tissue using a liquid/liquid extraction procedure with isoamylalcohol/n-hexane/borate buffer pH 9.3 (0.05/5/0.25 v/v/v). Chromatography was performed on a Polaris® (Varian Inc. Palo Alto, CA, USA) C18, 5 m, 50 mm×2 mm analytical column at a flow rate of 1,0 ml/min using acetonitrile/phosphoric acid buffer pH 2 (15/45 v/v) at 30°C. The range where the assay was linear (50 and 500 ug/L), the coefficient of variation was less than 10% and the accuracy was between 95 and 105% for both fluoxetine and fluvoxamine.

### Pathological examination

Mice were sacrificed at P20 and adulthood, and heart, lungs, liver, stomach, kidneys, intestines and spleen were dissected. Tissue was fixed in 4% paraformaldehyde, dehydrated in an ethanol series, embedded in paraffin. Paraffin-embedded sections (7 µm) were mounted on SuperFrost plus slides (Menzel Gläser), deparaffinated, stained with haematoxylin and eosin, and analyzed by two independent observers blinded for the treatment.

### Magnetic Resonance Imaging

The MRI scanning of the hearts was performed as described before [14].

### Autoradiography

P20 and adult brains were collected and immediately frozen on dry ice. Sections (20 µm) were cut and collected on SuperFrost Plus slides (Menzel Gläser). Brain sections were stored at −80°C until use. Sections were dried with a stream of air and washed for 20 min in Tris-HCl buffer (120 mM NaCl, 50 mM Tris-HCl (Sigma-Aldrich), pH 7.4) at room temperature. For specific binding to the serotonin transporter, sections were incubated for 1 hour with 1 nM [N-methyl-^3^H]-citalopram (77.0 Ci/mmol; Amersham Bioscience, UK) in Tris-HCl buffer at room temperature. Non-specific binding was determined by adding 10 µM of the selective serotonin transporter inhibitor, fluoxetine (Sigma-Aldrich) in addition to 1 nM [N-methyl-^3^H]-citalopram. Sections were then washed three times (1, 10 and 10 min, respectively) in ice cold Tris-HCl buffer and rinsed in ice cold water. Sections were dried with a stream of air and exposed to BAS-TR2040 phosphoimaging plates (Fuji Imaging Plates) for 2 weeks at room temperature. Autoradiographic BAS-TR2040 imaging plates were scanned using the FLA-5000 imaging system (Fuji) and quantitative analysis was performed using the AIDA Image Analyzer Software (Raytest). Serotonin transporter binding was analyzed in the dorsal raphe nucleus of mice at P20 and P90 treated with saline (N = 10), fluvoxamine (N = 10) and fluoxetine (N = 4).

### Elevated Plus Maze

The elevated plus maze (black Plexiglas) was elevated 100 cm above floor level and consisted of two open and two closed arms (30×5 cm). All arms radiated from a common central open square (5×5 cm). The floor of the enclosed arms was the same size as the open, but these arms had side walls of 15 cm high. Each mouse was placed in the plus-maze facing an open arm and was allowed to explore freely the plus-maze for 10 min. All the experiments were performed under normal ambient overhead lighting and were carried out during the light phase of the cycle, between 13:00 and 18:00 h. Each experimental session was recorded by videotracking software (Ethovision, Noldus Information Technology, The Netherlands). Between trials, the apparatus was thoroughly rinsed with 70% ethanol and dried with clean towels. The following parameters were obtained: total distance moved, velocity, entries into open and closed arms, time spent on open and closed arms and distance moved on open and closed arms. Mice were tested at P20 and adulthood.

### Large Open Field

The open field consisted of a dark-gray PVC cylinder with a diameter of 80 cm and 30 cm in height. The open field was divided in two parts, a central area with a diameter of 55 cm and an outer ring. Adult mice were individually transported from the adjacent room to the experimental room and immediately placed near the wall, in the outer ring of the open field. All the experiments were performed under normal ambient overhead lighting and were carried out during the light phase of the cycle, between 13:00 and 18:00 h. Locomotor activity was recorded by videotracking software (Ethovision, Noldus Information Technology, The Netherlands) for 30 min. Between trials, the apparatus was thoroughly rinsed with 70% ethanol and dried with clean towels. The following parameters were obtained: total distance moved, velocity, time spent in the center and in the outer ring and distance moved in the center and in the outer ring.

### Novelty suppressed feeding test

Adult mice were weighed and food was removed from the cage, while water remained available ad libitum. Twenty-four hours after food restriction, mice were transferred to the testing room, placed in a novel arena with in the center a petridish containing a pre-weighed quantity of food pellets. Each subject was placed in the corner of the testing area, and the latency to feed, time spent feeding, and total food consumption were recorded over 10 min. All the experiments were performed under normal ambient overhead lighting and were carried out during the light phase of the cycle, between 13:00 and 18:00 h.

### Statistics

Data of body weight, Wt/r ratios, 5-HTT binding and behavioral performance were analyzed using an one-way analysis of variance (ANOVA), followed by Bonferroni multiple comparison test when appropriate. Within-group comparisons were performed using Student's paired t test. P<0.05 was considered significant.

## Results

### Placental transfer of fluoxetine and fluvoxamine is comparable between mouse and human

**Figure 1:**
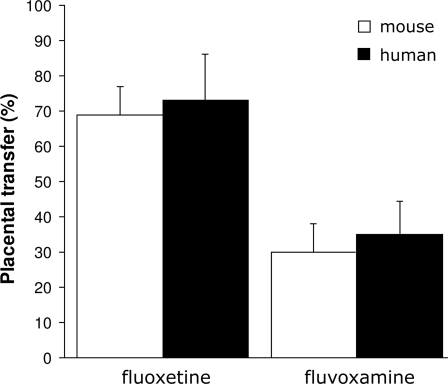
Placental transfer of fluoxetine and fluvoxamine in mouse (white bars) and human (black bars) presented as mean±S.E.M. Fluoxetine showed a high placental transfer in both mice (69%; N = 4) and human (73%; N = 6), while fluvoxamine has a low placental transfer in mice (30%; N = 4) and human (35%; N = 2).

The level of fetal exposure to certain SSRIs may influence the effects of SSRIs on fetal development, and may differ due to specific placental transfer efficiency. Therefore, we determined the placental transfer of two commonly used SSRIs (fluoxetine and fluvoxamine) in mouse and human (Fig. 1). A relatively high placental transfer of fluoxetine was observed in both mice (69%, N = 4) and humans (73%, N = 6). In contrast, a low placental transfer of fluvoxamine was found in both mice (30%, N = 4) and humans (35%, N = 2). These data indicate that fetal exposure of fluoxetine and fluvoxamine is comparable between mouse and human. Furthermore, fluoxetine-treated fetuses are exposed to higher levels SSRIs as compared to fluvoxamine-treated fetuses.

### Prenatal SSRI treatment does not affect the body weight of the offspring

Since it has been reported that prenatal SSRI exposure can affect body weight in humans [12], [13], we determined the body weight of prenatal fluoxetine-and fluvoxamine-treated offspring in mice at various stages (Table 1). Four stages were included in the analysis based on drug exposure and fetal development: during treatment (E18), in the withdrawal period (P3), at the end stage of brain development (P20) and in adulthood (P90). The data show that prenatal exposure to fluoxetine and fluvoxamine did not affect the body weight of the offspring at the stages analyzed. Body weights of males and females were not significantly different at E18, P3 and P20 (data not shown) and were combined for these stages.

**Table 1:** Body weight during postnatal development and between treatment groups.

*Drug*	E18	P3	P20	Adult	
				Male	female
Saline	1.21±0.11 (n = 36)	1.96±0.22 (n = 32)	8.40±0.97 (n = 32)	30.35±1.96 (n = 15)	22.34±0.95 (n = 17)
Fluvoxamine	1.15±0.09 (n = 37)	1.96±0.20 (n = 34)	8.21±0.83 (n = 34)	29.88±1.33 (n = 18)	22.00±0.80 (n = 16)
Fluoxetine	1.23±0.11 (n = 33)	1.93±0.12 (n = 26)	8.45±0.84 (n = 6)	30.88±1.93 (n = 4)	23.14±0.47 (n = 2)

### Prenatal fluoxetine exposure causes severe dilated cardiomyopathy resulting in a decreased survival rate

**Figure 2:**
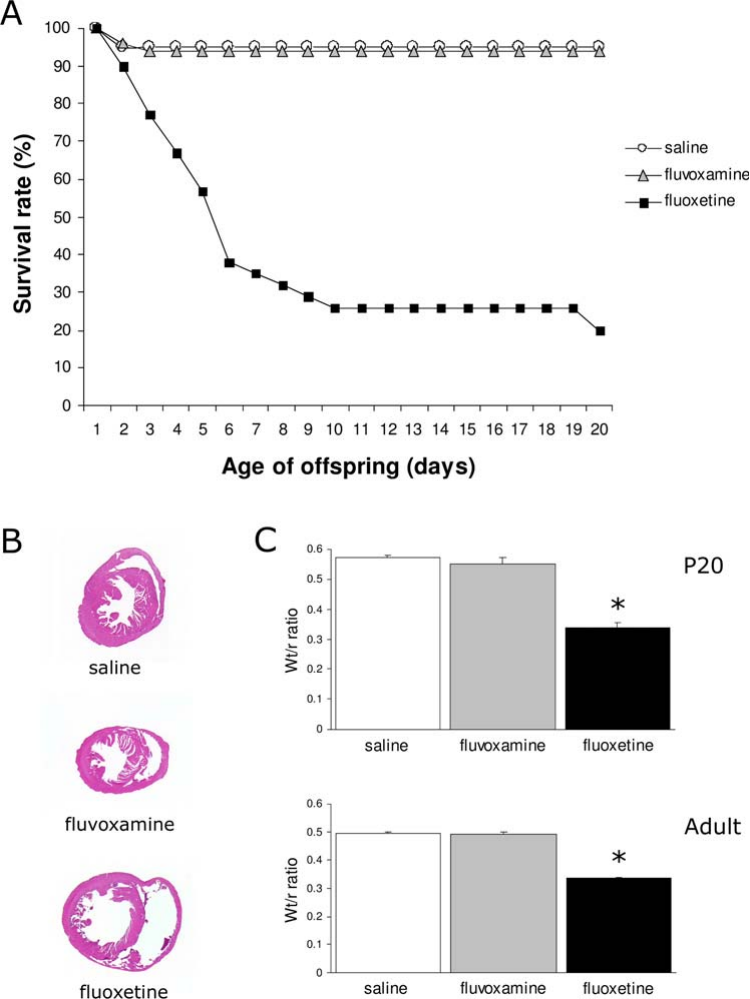
(A) Survival rates of mice prenatally treated with saline (white circle), fluvoxamine (gray triangle) or fluoxetine (black square) from 1 to 20 days after birth. 81% of the fluoxetine-treated offspring died within 20 days after birth. (B) HE-staining of a horizontal section of the hearts showed dilated cardiomyopathy in fluoxetine-treated offspring. (C) Wt/r ratios of the left ventricle presented as mean±S.E.M for groups treated with saline (white bars), fluvoxamine (gray bars) and fluoxetine (black bars) at P20 (N = 6 for saline and fluvoxamine, N = 4 for fluoxetine) and adulthood (N = 5 for saline and fluvoxamine, N = 3 for fluoxetine). Wt/r = wall thickness/radius, * p<0.01.

Surprisingly, prenatal fluoxetine exposure dramatically decreased the survival rate during the preweaning period (Fig. 2A). The majority of the fluoxetine-treated offspring died within 6 days after birth (62%), while 19% of the offspring died between P7 and P20. Notably, after P20 all the remaining pups survived until adulthood. In contrast, no differences in survival rate were observed in the fluvoxamine exposed offspring. Thus, fluoxetine treatment showed a dramatic effect on the mortality of the offspring (81%), while fluvoxamine treatment did not affect the survival rate of the offspring.

To investigate whether the mortality was due to maternal aspects, fetal aspects or a combination of both, we performed cross-fostering experiments. In these experiments only the fluoxetine-treated offspring died, with no difference in mortality rate between treated and untreated mothers, suggesting that the high mortality rate was due to fetal aspects. To obtain an indication of the possible cause of death, fluoxetine-, fluvoxamine- and saline-treated mice were sacrificed at P20 and adulthood for histopathological examination of the organs. No abnormalities were observed in the lungs, liver, stomach, kidneys, intestines and spleen (data not shown). However, the hearts of fluoxetine-treated animals were enlarged at both stages, indicating dilated cardiomyopathy, while the hearts of fluvoxamine-treated mice were normal compared to the saline-treated hearts (Fig. 2B). To generate a quantitative measure for the dilated cardiomyopathy, the wall thickness of the left ventricle and the radius of the left ventricular cavity were measured, and wall thickness/radius (Wt/r) ratios were calculated for all groups (Fig. 2C). Fluoxetine-treated offspring showed a significantly decreased Wt/r ratio at both P20 (40% decrease; N = 4; p<0.01) and adult stage (32% decrease; N = 3; p<0.01) as compared to saline-treated offspring (N = 6 for P20; N = 5 for adult). No deviations of these parameters were observed in fluvoxamine-treated offspring at either P20 (N = 6) or adult as compared to the saline controls (N = 5). A decrease of the Wt/r ratio was found between P20 and adulthood in both the saline (p<0.01) and fluvoxamine group (p<0.07), indicating changes in heart morphology during aging. However, these aging effects were not observed in the fluoxetine-treated group (p = 0.84). In agreement with these findings, in-vivo MRI experiments showed a increased left ventricular cavity and a decreased wall thickness in the fluoxetine-exposed offspring compared to the saline-exposed offspring, with no alterations after prenatal fluvoxamine treatment (supplemental Movie S1). Taken together, these data clearly show that prenatal fluoxetine exposure severely affects heart development, resulting in a high mortality rate of the offspring, whereas fluvoxamine treated offspring is unaffected.

### SSRI exposure during fetal development induces long-term alterations in the serotonin system

**Figure 3:**
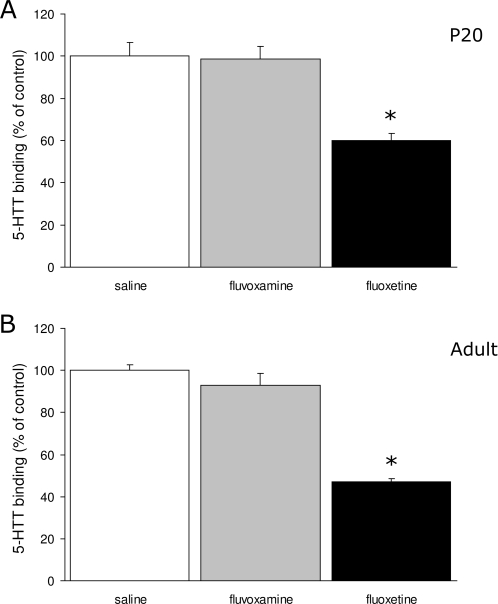
Binding of 5-HTT in the raphe nucleus presented as percentage of control±S.E.M for groups treated with saline (white bars), fluvoxamine (gray bars) and fluoxetine (black bars) at P20 (A) (N = 6 for saline and fluvoxamine, N = 4 for fluoxetine) and adulthood (B) (N = 6 for saline and fluvoxamine, N = 3 for fluoxetine). * p<0.01.

Since SSRIs bind specifically to the 5-HTT, 5-HTT levels may be permanently altered as a consequence of SSRI treatment during fetal brain development. To study the long-term effects of prenatal fluoxetine and fluvoxamine exposure on the 5-HTT levels in the brain region where the serotonergic cell bodies are located (raphe nucleus), autoradiography using [N-methyl-^3^H]-citalopram was performed on P20 and adult brain tissue (Fig. 3). After prenatal exposure to fluoxetine, 5-HTT binding decreased significantly in the raphe nucleus at P20 (40%; N = 4; p<0.01) and adult stage (53%; N = 3; p<0.01) as compared to saline-treated offspring (N = 6 for P20 and adult). No statistical significant reductions of 5-HTT binding in the raphe nucleus were observed after prenatal fluvoxamine exposure at either P20 (N = 6) or adult (N = 6). Taken together, these data indicate that prenatal fluoxetine exposure changes the 5-HT homeostasis as measured through 5-HTT expression levels, whereas fluvoxamine exposure does not.

### Prenatal SSRI exposure results in altered behavior at adulthood

**Figure 4:**
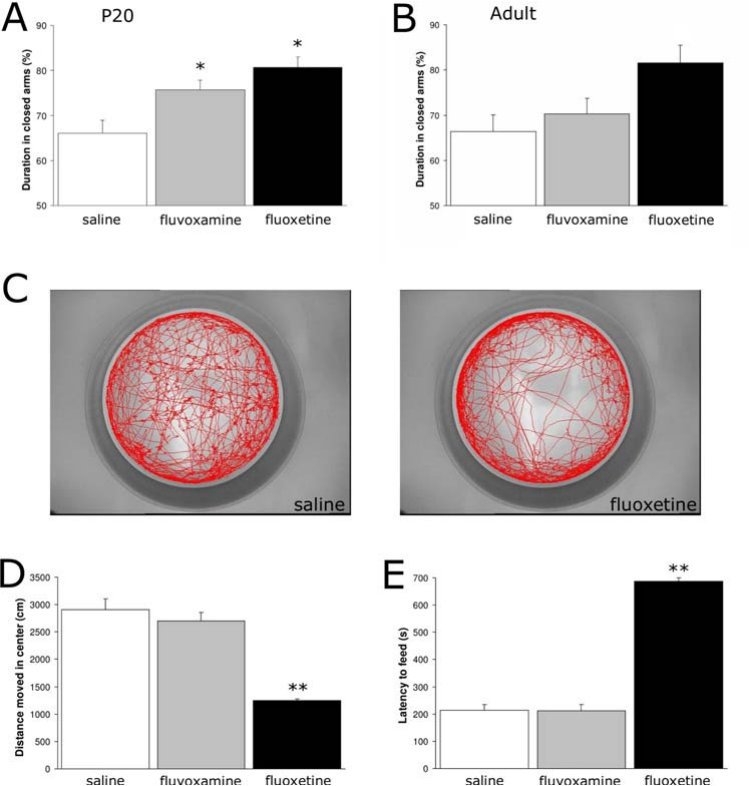
Behavioral data of the elevated plus maze (A, B), open field (C, D) and the novelty suppressed feeding test (E) presented as mean±S.E.M. All behavioral test were performed with 3 groups: saline (white bars), fluvoxamine (gray bars) and fluoxetine (black bars). Panel C shows a typical example of a walking pattern in the open field (30 min) of a saline- and a fluoxetine-treated mouse. * p<0.05; ** p<0.01.

To investigate whether prenatal SSRI exposure and the above described fluoxetine-induced changed 5-HT homeostasis influences anxiety related behavior, we tested mice in an elevated plus maze, an open field and in a novelty suppressed feeding test. In the elevated plus maze, no alterations were observed in exploratory behavior, since SSRI-treated mice showed no difference in total distance moved, velocity and time spent ambulating compared to saline-treated mice at both P20 and adulthood (data not shown). However, the duration in the closed arms was significantly increased at P20 after both fluvoxamine (N = 13; p<0.05) and fluoxetine treatment (N = 3; p<0.05) as compared to saline-treated mice (N = 13; Fig. 4A). At adulthood, no significant differences were observed, although fluoxetine-treated offspring did spend more time in the closed arms (Fig. 4B). Next, mice were tested in an open field at adulthood (Fig. 4C, D). Figure 4C demonstrates a typical example of walking patterns of a saline- and a fluoxetine-treated mouse. During 30 min, saline-treated mice explored the whole arena, while fluoxetine-treated mice stayed close to the wall in the outer ring. No differences were measured in the total distance moved between the groups (data not shown), but fluoxetine-treated mice showed a significant decrease in distance moved in the central area (N = 3; p<0.01; Fig. 4D) as compared to the saline-treated group (N = 12). Fluvoxamine exposed mice (N = 12) showed no alterations in the open field compared to the control group. To assess the effects of prenatal SSRI exposure on emotional functioning, we performed a novelty suppressed feeding test. This test is thought to demonstrate depression- and anxiety related behaviors, since animal models of anxiety and depression are abnormal in this test [15], [16]. Weight loss during food restriction and latency to feed in the home cage were not different between groups (data not shown), which indicates no alterations in motivational factors. However, fluoxetine-treated mice showed a 3-fold increase in the latency to feed (N = 3; p<0.01) as compared to saline-treated mice (N = 13), while fluvoxamine-treated mice showed the same latency as compared to the control group (N = 12; Fig. 4E). Taken together, these data show that prenatal fluoxetine exposure results in depression- and anxiety related behavior at adulthood, while no effects were observed after prenatal fluvoxamine treatment.

### Heart failure causing increased mortality after prenatal fluoxetine exposure is dose-dependent

**Figure 5:**
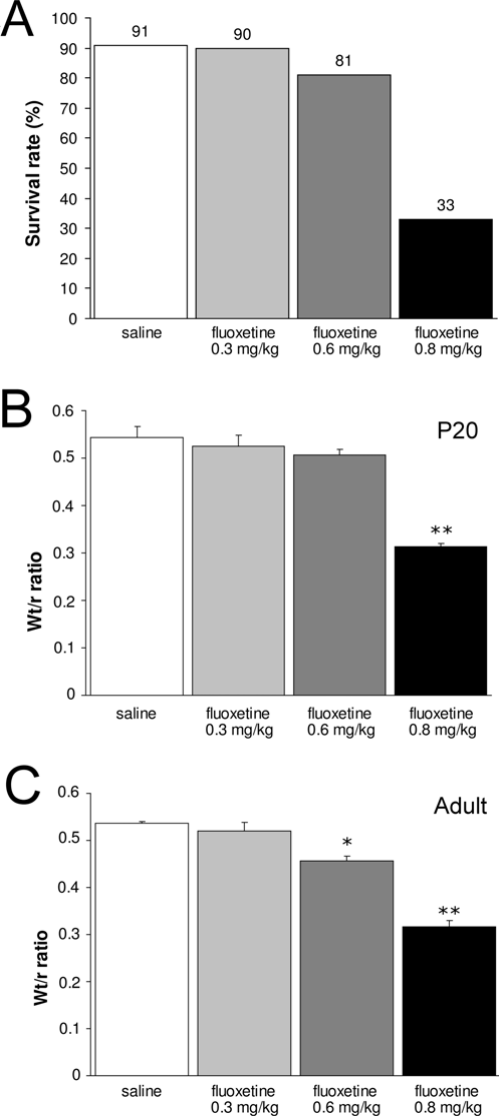
Survival rate and heart pathology after prenatal exposure to different doses of fluoxetine. (A) Survival rates of mice prenatally exposed to saline (white bars), 0.3 mg/kg/day fluoxetine (light gray bars), 0.6 mg/kg/day fluoxetine (dark gray bars) and 0.8 mg/kg/day fluoxetine (black bars) measured at adulthood. 67% of the offspring exposed to the highest dose fluoxetine (0.8 mg/kg/day) died during the preweaning period as compared to 9% of the saline-treated offspring. Wt/r ratios of the left ventricle are presented mean±S.E.M for groups treated with saline (white bars; N = 5), 0.3 mg/kg/day fluoxetine (light gray bars; N = 5), 0.6 mg/kg/day fluoxetine (dark gray bars; N = 5) and 0.8 mg/kg/day fluoxetine (black bars; N = 5) at P20 (B) and adulthood (C). Wt/r = wall thickness/radius, * p<0.05; ** p<0.01.

To determine whether the mortality rate after prenatal fluoxetine treatment (0.8 mg/kg/day) is dose-dependent, offspring of mice exposed to different concentrations of fluoxetine (0.3, 0.6 and 0.8 mg/kg/day) during pregnancy were studied. Only mice prenatally exposed to the highest fluoxetine concentration (0.8 mg/kg/day) displayed a dramatic decrease in survival rate measured at adult stage (Fig. 5A). In agreement with our previously described findings (Fig. 2), prenatal fluoxetine-treated mice (0.8 mg/kg/day) dramatically decreased the survival rate during the preweaning period. Offspring exposed to a lower dose fluoxetine (0.6 mg/kg/day) demonstrated a small decrease in survival rate, whereas mice exposed to the lowest dose fluoxetine (0.3 mg/kg/day) showed survival rates identical as saline-treated mice. These data clearly show a dose-dependent effect of fluoxetine on the survival rate of the offspring. In line with these findings, we observed alterations in the Wt/r ratio at both P20 and the adult stage (Fig. 5B and C, respectively). At P20, mice prenatally exposed to the highest fluoxetine concentration (0.8 mg/kg/day) demonstrated a dramatic decrease in Wt/r ratio (43% decrease; N = 5; p<0.01) compared to saline-treated mice (N = 5), whereas the Wt/r ratios of the lower doses (0.6 mg/kg/day (N = 5) and 0.3 mg/kg/day (N = 5) were similar as saline-treated offspring (Fig. 5B). The highest dose fluoxetine (0.8 mg/kg/day) had a similar effect on the Wt/r ratio when measured at the adult stage (40% decrease; N = 5; p<0.01; Fig. 5C) as compared to saline-treated offspring (N = 5). In addition, 0.6 mg/kg/day fluoxetine had a significant effect on the Wt/r ratio in adulthood (15% decrease; N = 5; p<0.05; Fig. 5C). The lowest concentration fluoxetine (0.3 mg/kg/day; N = 5) did not effect the Wt/r ratio in adulthood. Taken together, these findings indicate that prenatal fluoxetine exposure affects heart development in a dose-dependent manner, resulting in a fluoxetine dose-dependent mortality rate of the offspring.

### Long-term alterations in behavior and in the serotonin system after prenatal fluoxetine exposure are dose-dependent

**Figure 6:**
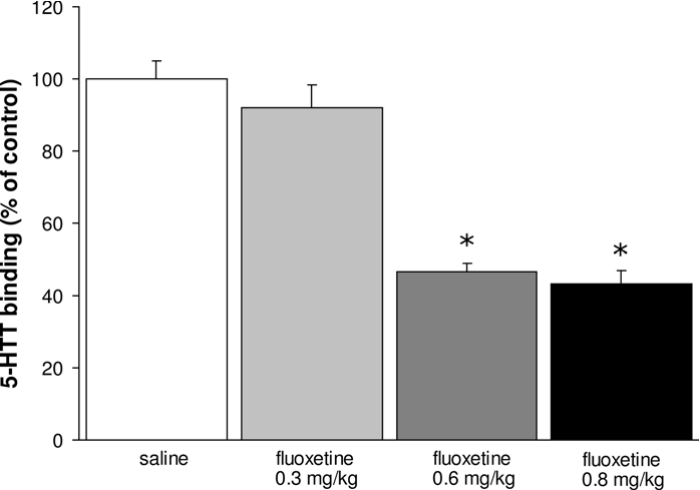
Binding of 5-HTT in the raphe nucleus at adulthood expressed as percentage of control±S.E.M. for groups treated with saline (white bars), 0.3 mg/kg/day fluoxetine (light gray bars), 0.6 mg/kg/day fluoxetine (dark gray bars) and 0.8 mg/kg/day fluoxetine (black bars). * p<0.01.

To determine whether the decreased 5-HTT levels in the raphe nucleus after prenatal fluoxetine treatment (0.8 mg/kg/day) are dose-dependent, autoradiography using [N-methyl-^3^H]-citalopram was performed on adult brain tissue of mice prenatally exposed to different concentrations of fluoxetine (0.3, 0.6 and 0.8 mg/kg/day; Fig. 6). 5-HTT binding was significantly decreased in the offspring exposed to 0.8 (N = 6; p<0.01) and 0.6 mg/kg/day fluoxetine (N = 6; p<0.01) as compared to the saline-treated offspring (N = 6). In contrast, offspring exposed to the lowest dose fluoxetine (0.3 mg/kg/day) did not show alterations in the 5-HTT levels (N = 6) as compared to the saline-treated group. Taken together, these data clearly show that long-term alterations in the serotonin system after fluoxetine exposure are dose-dependent.

**Figure 7:**
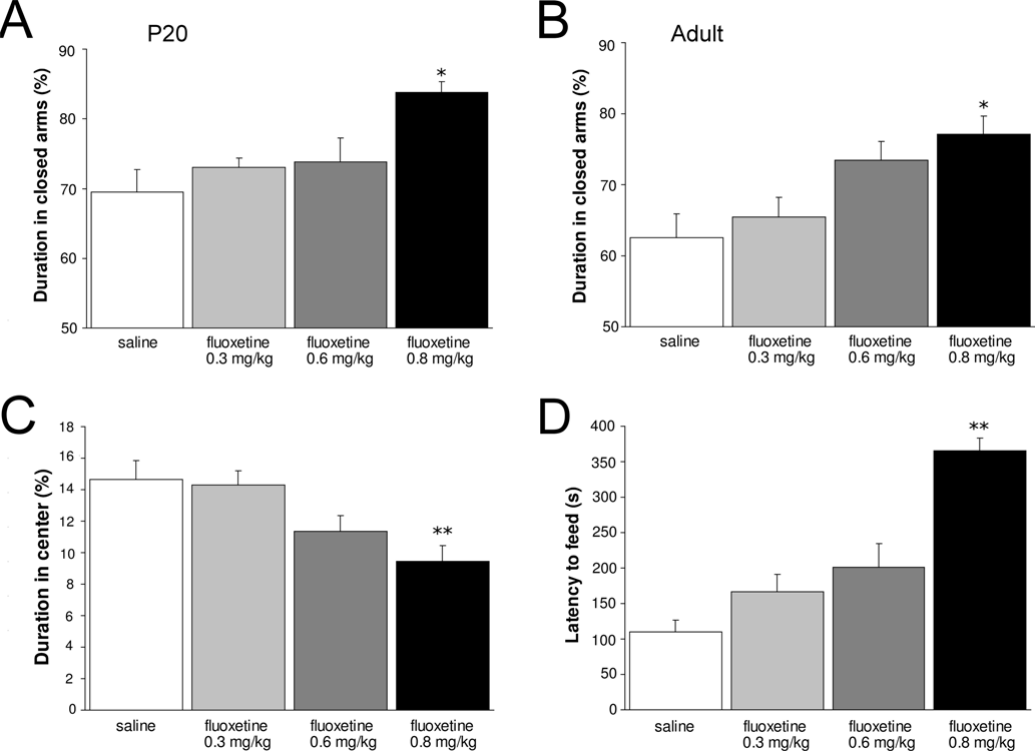
Behavioral data of the elevated plus maze (A, B), open field (C) and the novelty suppressed feeding test (D) presented as mean±S.E.M. for groups treated with saline (white bars), 0.3 mg/kg/day fluoxetine (light gray bars), 0.6 mg/kg/day fluoxetine (dark gray bars) and 0.8 mg/kg/day fluoxetine (black bars). * p<0.05; ** p<0.01.

To determine whether the long-term effects of prenatal fluoxetine exposure (0.8 mg/kg/day) on depression- and anxiety related behavior are dose-dependent, we tested mice prenatally exposed to different concentrations of fluoxetine (0.3, 0.6 and 0.8 mg/kg/day; Fig. 7) in an elevated plus maze at P20 (Fig. 7A) and adulthood (Fig. 7B). No alterations were observed in exploratory behavior, since all fluoxetine-treated groups showed similar total distances moved, velocity and time spent ambulating compared to saline-treated mice (data not shown). The highest dose of fluoxetine (0.8 mg/kg/day) showed a significant increase in duration in the closed arms compared to the control group, both at P20 (N = 9; p<0.05) and adulthood (N = 5; p<0.05). The 0.3 mg/kg/day (N = 5) and 0.6 mg/kg/day (N = 5) fluoxetine groups were not significantly different from the saline-treated group, but a clear dose-response effect on the duration in the closed arms can be observed at adulthood. At P20, no dose-response effect could be found since 0.3 (N = 13) and 0.6 mg/kg/day (N = 10) showed the same effect on the offspring in the elevated plus maze as compared to the saline-treated mice (N = 12).

Next, adult mice were tested in an open field (Fig. 7C). No differences were measured in the total distance moved between the groups (data not shown), but offspring treated with the highest dose fluoxetine (0.8 mg/kg/day) showed a significant decrease in distance moved in the central area (N = 9; p<0.01) as compared to the saline-treated group (N = 9), which is in agreement with our previous findings (Fig. 4D). Although, the 0.3 and 0.6 mg/kg/day fluoxetine groups (N = 9) were not significantly different from the saline-treated group, a clear dose-response effect on the distance moved in the central area could be observed. Similar findings were observed in the novelty suppressed feeding test performed at adulthood (Fig. 7D). Weight loss during food restriction and latency to feed in the home cage were not different between groups (data not shown), which indicates no alterations in motivational factors. However, offspring treated with the highest dose fluoxetine showed a 3-fold increase in the latency to feed (N = 10; p<0.01) as compared to saline-treated mice (N = 10), which is in agreement with our previous findings (Fig. 4E). Similar as for the open field results, the 0.3 and 0.6 mg/kg/day fluoxetine groups (N = 10) were not significantly different from the saline-treated group (N = 10), but a clear dose-response effect could be detected. The 0.6 mg/kg/day fluoxetine group showed a tendency for increased latency to feed (p = 0.07) as compared to the saline-treated group. Taken together, these data clearly indicate a dose-dependent effect of fluoxetine on long-term depression- and anxiety related behavior.

## Discussion

To assess potential long-term effects of prenatal SSRI exposure, we administered clinical doses of fluvoxamine and fluoxetine to pregnant mice. Embryos were exposed to SSRIs between E8 and E18, when the 5-HTT is present [17], [18]. After prenatal fluoxetine exposure, we found a dose-dependent increase in neonatal mortality in the offspring, a permanent decreased 5-HTT binding and significant long-term alterations in depression- and anxiety related behavior.

In order to ensure the relevancy of this study for the human situation, we made a comparison of the fetal exposure of fluvoxamine and fluoxetine between mouse and human in terms of placental transfer. The results showed that the placental passage is similar between mouse and human, with respect to fluvoxamine and fluoxetine. Fluvoxamine displayed a relatively low placental transfer, while fluoxetine showed a relatively high placental transfer. Thus, fluoxetine-treated fetuses are exposed to higher levels compared to fluvoxamine-treated fetuses. Therefore, we hypothesized that offspring exposed to fluoxetine would have more possible long-term consequences compared to offspring exposed to fluvoxamine.

In the present study, no alterations were found in body weight between E18 and adulthood of both the fluvoxamine and fluoxetine exposed offspring, which is consistent with others who administered a therapeutic dose of SSRIs in mice [19]. However, our findings conflict with a report which describes that rats treated with high doses of fluoxetine during pregnancy have smaller pups with poorer weight gain [20]. In human, many studies have described lower birth weight after prenatal SSRI exposure [12], [13], [21]. Although, Chambers et al. [7] described a link between prenatal fluoxetine exposure and low birth weight that disappeared when maternal weight gain was controlled.

Surprisingly, the offspring exposed to high doses of fluoxetine showed a dramatic high level of mortality during the postnatal period, whereas mortality was low in the saline- and fluvoxamine-treated group. The cross-fostering experiments demonstrated that the effects of prenatal fluoxetine treatment on mortality were due to fetal aspects, which excludes maternal effects on the survival rate of the offspring. By histopathological examination and measuring the wall thickness/radius ratio, we observed that the majority of the fluoxetine-treated offspring died of heart failure, due to dilated cardiomyopathy. Dilated cardiomyopathy was also observed in mice lacking the serotonin 2B receptor, which is required for heart development [22], [23]. Nebigil et al. [22] also showed that the serotonin 2B receptor is required for 5-HT to regulate cardiovascular functions. This suggests that the 5-HT system is involved in heart development and that fluoxetine treatment during fetal development affects the heart, resulting in dilated cardiomyopathy. Major cardiac malformations have also been associated with prenatal SSRIs in human practice, although most of the cardiac malformations were observed after prenatal paroxetine treatment [4], [24], [25]. Fortunately, no association has been found between prenatal SSRI exposure and neonatal death in human practice. Differences in neonatal mortality between mouse and human can be explained by the neonatal care, which is excellent in humans but poor in mice. An alternative explanation is that, due to species differences in development, neonatal death in mice is similar to prenatal death in humans. Chambers et al. [7] observed that mothers exposed to fluoxetine had an increase in the incidence of miscarriages. Moreover, in a meta-analysis of clinical trials it was shown that prenatal SSRIs significantly increased the risk for spontaneous abortion [26], which is also observed in mice [27]. Since we found no effects of prenatal fluvoxamine exposure on the survival rate and the hearts of the offspring, fluvoxamine may be a safer SSRI during pregnancy compared to fluoxetine. Nevertheless, more research is necessary to exclude the effects of prenatal fluoxetine exposure on heart development in humans.

The observed changes in 5-HTT binding in the raphe nucleus at P20 and adulthood demonstrates that fluoxetine exposure during fetal development permanently changes the serotonin homeostasis, whereas prenatal fluvoxamine exposure did not. This data is in agreement with other reports, which showed that chronic treatment with fluoxetine downregulates the 5-HTT in adult rodents [28]–[30]. However, these animals were treated with SSRIs at adulthood. To our knowledge, this is the first study which describes the effects of prenatal SSRI treatment on 5-HTT density. Since 5-HTT appeared to be a critical regulator of emotional function, we have investigated whether these 5-HTT alterations resulted in changes of behavior. All behavioral experiments showed that fluoxetine exposed mice demonstrated depression- and anxiety-related behavior, whereas fluvoxamine exposed offspring showed no changes in behavior at adulthood compared to saline-treated offspring. These results are comparable to the behavioral data of the 5-HTT null mutant mice, which demonstrated a range of behavioral and neurophysiological abnormalities that resemble symptoms of mood and anxiety disorders [31]. To date, there have been insufficient long-term follow-up studies in human to demonstrate effects of prenatal SSRI on the risk of developing affective disorders in the offspring. Further investigation of the long-term consequences of fetal exposure to SSRIs, as well as the mechanisms involved, are required for a better understanding of the impact of SSRIs on development of the offspring.

Decreasing the dose of fluoxetine in a dose-response experiment, with therapeutic doses ranging from 0.3 till 0.8 mg/kg/day, resulted in higher survival rates and less dramatic effects on the long-term behavior in the offspring. Interestingly, the effects observed after administration of 0.8 mg/kg/day were less severe when a lower dose (0.6 mg/kg/day) was used, and completely diminished at the lowest dose administered (0.3 mg/kg/day). Noteworthy, the sub-maximal clinical dose of fluoxetine (0.6 mg/kg/day) still resulted in heart failure and behavioral pathology in the offspring. These results indicate that the effects of prenatal fluoxetine treatment on the fetus are dose-dependent.

The present findings demonstrate that prenatal fluoxetine treatment has dramatic effects on the survival rate of the offspring, alter the 5-HT system homeostasis at adulthood and indicate that fluoxetine exposed mice are more vulnerable to anxiety disorders at adulthood. Ultimately, clinical studies will be required to determine whether our findings have applicability to the risks for anxiety or affective disorders in humans. Two recent papers on this subject argue that it is safe to use antidepressants during pregnancy [32], [33]. Interestingly, the selection of patients was very restricted. Only pregnant women were included that used SSRIs in the first trimester. Although this is fine for general teratogenic effects of SSRIs, it is not appropriate to evaluate the effects of the pharmacological activity of SSRIs during development. It has been shown that the human serotonin system is developing in week 14–16 [34]. This would indicate that pharmacological intervention of the serotonin system by SSRIs in the infant is only effective after the first trimester. Taken together, we would argue that a clear study in humans about the relationship between SSRI intake and exposure of the infant, through placenta transfer efficiency parameters, is needed together with a broad behavioral assessment of the infant after the withdrawal effects.

Based on our findings in mice, prenatal fluvoxamine has no long-term consequences on the offspring in the clinical dose used, suggesting that fluvoxamine may be a safer antidepressant drug during pregnancy compared to fluoxetine. However, our data indicate that more detailed and specific follow-up studies in humans are required, since irreversible long-term adverse effects of SSRI treatment may be observed later in life. Our findings stress that it is important to be restrictive with prenatal fluoxetine administration, and that fluvoxamine may be the preferred SSRI during pregnancy.

## Supporting Information

Movie S1 MRI data of living heart of adult mice exposed prenatally to saline (ms51a), fluoxetine (ms28a) and fluvoxamine (no. ms46a). Shown is a movie (swf format) in which one heart beat is scanned in 10 frames. An axial viewpoint is taken from the initial volumetric data. The increased radius of the heart lumen can clearly be observed in the fluoxetine exposed animal (ms28a).
(0.29 MB SWF)
