# Correction: Modulation of Serotonin Transporter Function during Fetal Development Causes Dilated Heart Cardiomyopathy and Lifelong Behavioral Abnormalities

**DOI:** 10.1371/annotation/71abed9d-9ee9-4be0-a663-0d469750e13a

**Published:** 2009-09-09

**Authors:** Cornelle W. Noorlander, Frederique F. T. Ververs, Peter G. J. Nikkels, Cees J. A. van Echteld, Gerard H. A. Visser, Marten P. Smidt

The following text should be included in the Materials and Methods section as explanation of human data:

Human Data

The analysis of human maternal and infant cord blood was conducted within the ongoing OAZE study (trial registration: Current Controlled Trials number: ISRCTN25383361). The OAZE study is a prospective observational study on the effects of antidepressant use during pregnancy on mother and child, including pregnant women who visit the obstetrical outpatient clinic and are using SSRIs. The study was approved by the Central committee on Research Involving Human Subjects (CCMO) and the local Medical Ethical Committee of the University Medical Center Utrecht. A written informed consent was obtained from each subject before entering the study. Fetal drug exposure is studied by analyzing a maternal blood sample and an umbilical cord blood sample collected immediate after delivery. Samples are stored at -20°C till analysis. We reported the results of the first participants of the OAZE study (fluoxetine n=6 and fluvoxamine n=2) that were available. We analyzed the plasma levels of fluoxetine and fluvoxamine in maternal plasma and infant cord blood, using our HPLC-method which was also validated for human plasma.

